# Carcinoma Cervix Leading to Ichthyosis Uteri: A Rare Case Report

**DOI:** 10.1007/s13224-021-01472-3

**Published:** 2021-04-28

**Authors:** Komal Vijaywargiya, Namrata Kachhara, Qutbuddin Chahwala, Aayushi Ruia

**Affiliations:** 1 P.C. Sethi Hospital, 5/5 , Yeshwant Niwas Road, Rani Sati Colony, Indore, M.P India; 2 Medanta Hospital, Indore, India; 3 CHL Hospital, Indore, India

## Abstract

Ichthyosis uteri is an exceedingly rare condition in which the entire surface of the endometrium is replaced by stratified squamous epithelium. It is a benign lesion and its association with benign and malignant conditions has been reported in the literature (Bewtra, Xie, Hunter, Jurgensen (Arch Pathol Lab Med 29:e124–e125, 2005)). Originally described as an endometrial response to iatrogenically introduced caustic substances, similar changes have since been described in association with a variety of inflammatory conditions of the endometrium. It is concluded that a squamous cell carcinoma of the cervix extended proximally into the endometrium (Bagga, Jaswal, Datta, Mahajan (Indian J Pathol Microbiol 51:267–268, 2008)). The possibility of significant cervical pathology should be considered when plaques of squamous epithelium with low-grade dysplastic changes are identified in an endometrial biopsy or curettage.

**Supplementary Information:**

The online version contains supplementary material available at 10.1007/s13224-021-01472-3.

## Introduction

The term ichthyosis uteri is used when there is widespread replacement of the surface endometrium by keratinized stratified squamous epithelium [1]. It is considered a benign lesion but its association with malignancy has been reported in the literature [2]. We present a case report of ichthyosis uteri detected after Wertheim’s hysterectomy of a case presenting with pyometra.

## Case Report

A 65-yr-old woman, P8L8, all normal vaginal deliveries, who had menopause 11 yrs back presented to us with chief complaints of pain in lower abdomen and postmenopausal white discharge per vaginum since 7–8 months.

While her blood counts and urine examination were normal, MRI showed enlarged uterus of 16.1x10.4x13.5 cms with isointense endometrial collection of around 14.4x9.4x12.7 cms, approximately 860 cc. Her cervical biopsy revealed well-differentiated squamous cell carcinoma of cervix. Her chest CT scan and echocardiography were normal. Upon obtaining a valid written consent she was scheduled for pyometra drainage and followed by Wertheim’s hysterectomy. Her uterine specimen along with bilateral adnexa, pelvic lymph nodes were sent for histopathological examination. Histopathology report revealed squamous cell carcinoma in situ of cervix with dysplastic epithelium replacing entire endometrial cavity with bilateral reactive lymph nodes.

## Discussion

The term "ichthyosis uteri" was initially coined in 1885 by Zeller to refer to extensive squamous metaplasia of the surface endometrium following iatrogenically introduced caustic substances such as formalin or iodine [2]. Since that initial report, the term "ichthyosis uteri" and the phenomenon it describes have become well accepted but has been used only sporadically in the literature. The case reported herein is a cervical squamous cell carcinoma associated with extensive ichthyosis uteri-changes of the endometrium that, additionally, had superimposed low-grade dysplastic changes. This composite of findings may be explained in two, somewhat mutually exclusive ways: The first and most plausible explanation, and which formed the basis of the clinical diagnosis actually rendered, is that a squamous cell carcinoma originated in the cervix and the associated HPV extended proximally, colonizing a preexisting ichthyosis uteri. Due to the distinct rarity of this composite of findings, it is hypothesized that the immunocompromised state of the patient contributed to this process [1]. The second potential explanation is that within a background of extensive ichthyosis uteri, a squamous cell carcinoma developed in the lower uterine segment.

It is rarer compared to squamous metaplasia. Chronic trauma, repair, irritation, inflammation, foreign material, and estrogenic effects have all been implicated. The etiology of endometrial keratinization is not well understood. This rare pathology has been seen in association with benign conditions like tuberculous endometritis, puerperal endometritis, endometrial polyps, hyperplasia, and squamous papilloma and with pyometra as a result of cervical stenosis [3]. Hence, when ichthyosis is seen on endometrial curettage, one has to look for associated benign pathologies, especially tuberculosis, endometrial polyps, and hyperplasia.

According to some investigators, ichthyosis lacks malignant potential. However, dysplastic and anaplastic changes in the squamous epithelium have been reported, which may predispose to the rare endometrial squamous carcinoma in postmenopausal women [1].

The main differential diagnosis to be considered before making a diagnosis of pure ichthyosis is the extension of squamous carcinoma of the cervix into the endometrial cavity. The primary tumor in such a case would have an infiltrating rather than a polypoid morphology [2]. The extension of well-differentiated squamous carcinoma from the cervix can be distinguished from ichthyosis by detailed examination of the lower genital tract, by the presence of koilocytic changes and the presence of dysplastic changes in the squamous epithelium which favors a diagnosis of squamous carcinoma extension from cervix [2].

Although the number of cases of ichthyosis uteri associated with malignancies is disproportionately high relative to the overall number of cases reported, this is likely a bias created by the reporting of individual case reports. In our opinion, there is insufficient evidence to suggest that uncomplicated (i.e., non-dysplastic) ichthyosis uteri has any intrinsic neoplastic potential [2].

## Consent

Written informed consent was obtained from the patient for publication of this case report and accompanying images.

Informed consent from patient regarding use of her data for medical studies / research was taken.

**Figure:**
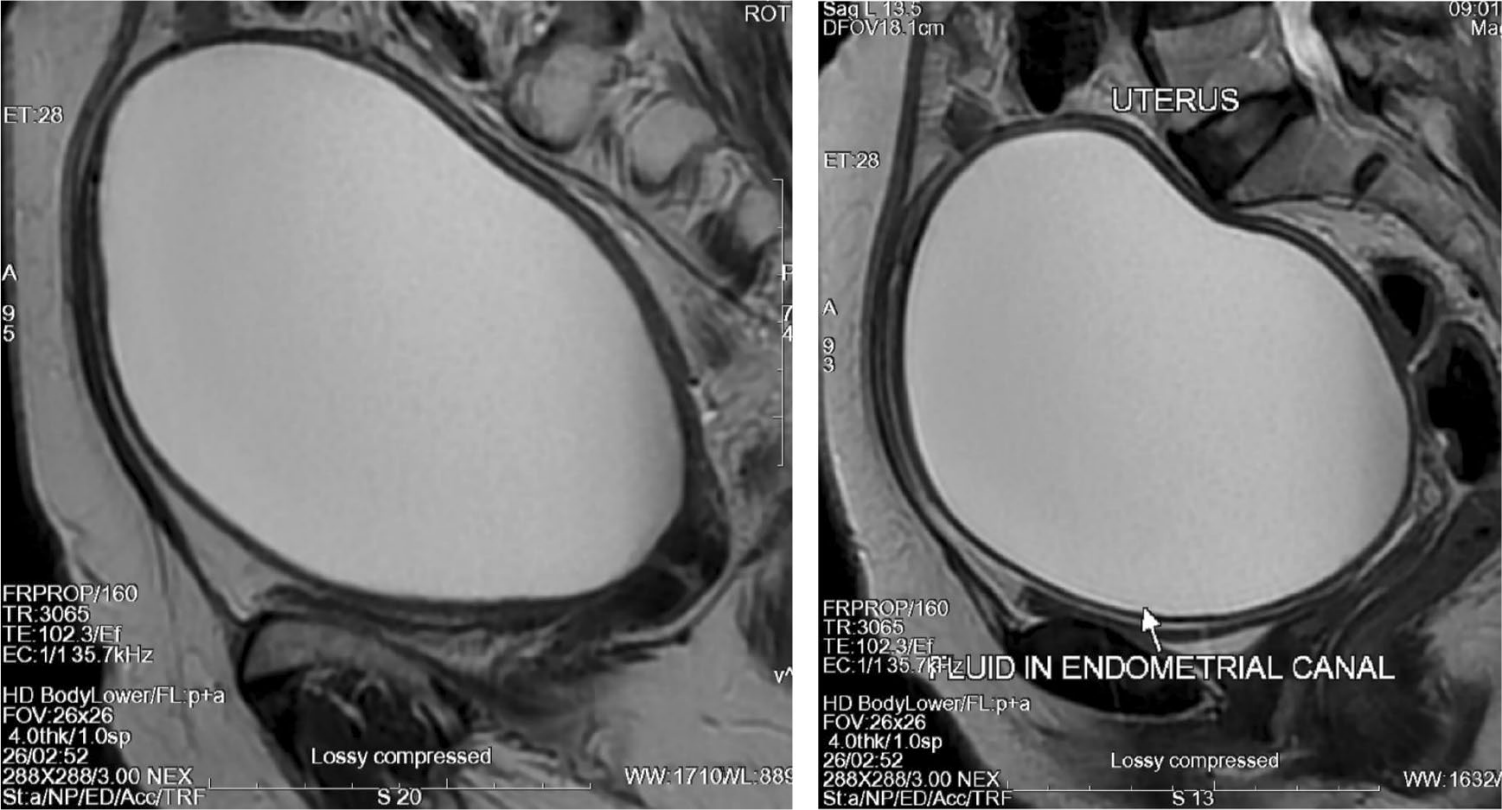
T2W MRI images showing enlarged uterus with large volume of high density fluid within endometrial cavity

**Figure:**
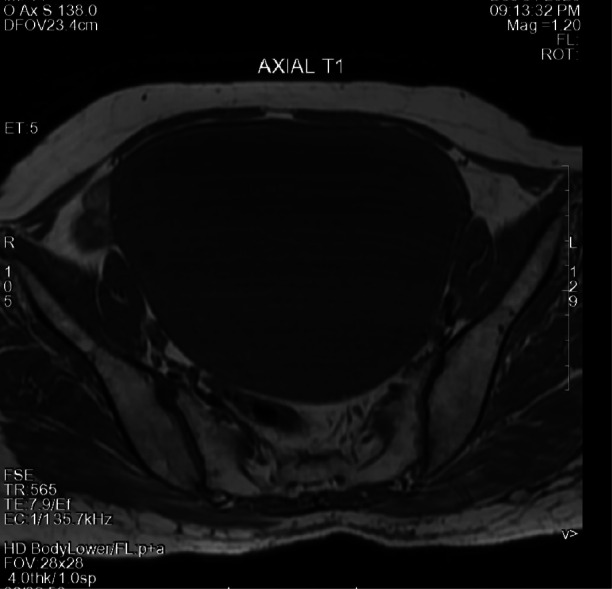
MRI axial view of the same

**Figure:**
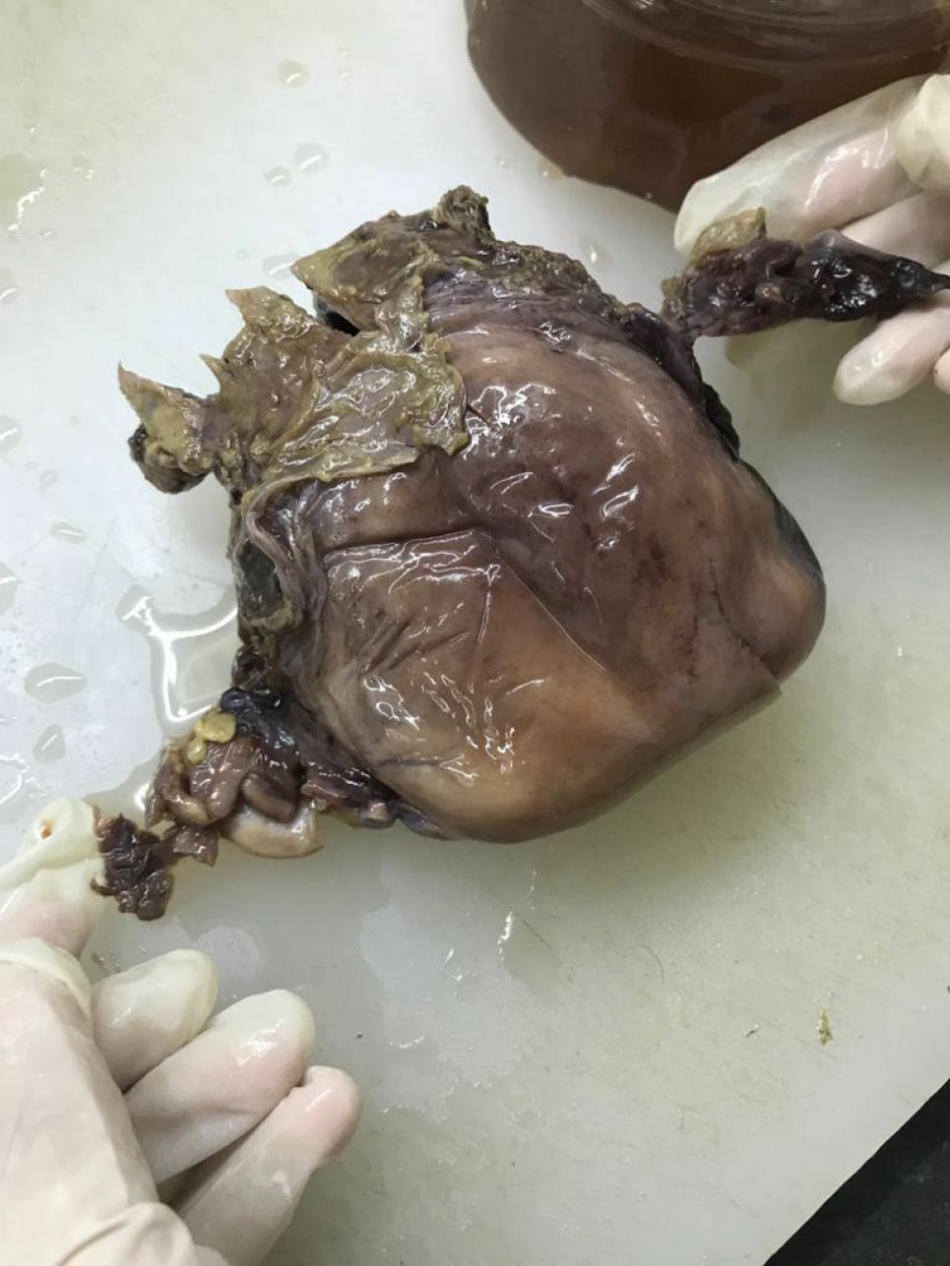
Gross specimen of uterus with its bilateral adnexa

**Figure:**
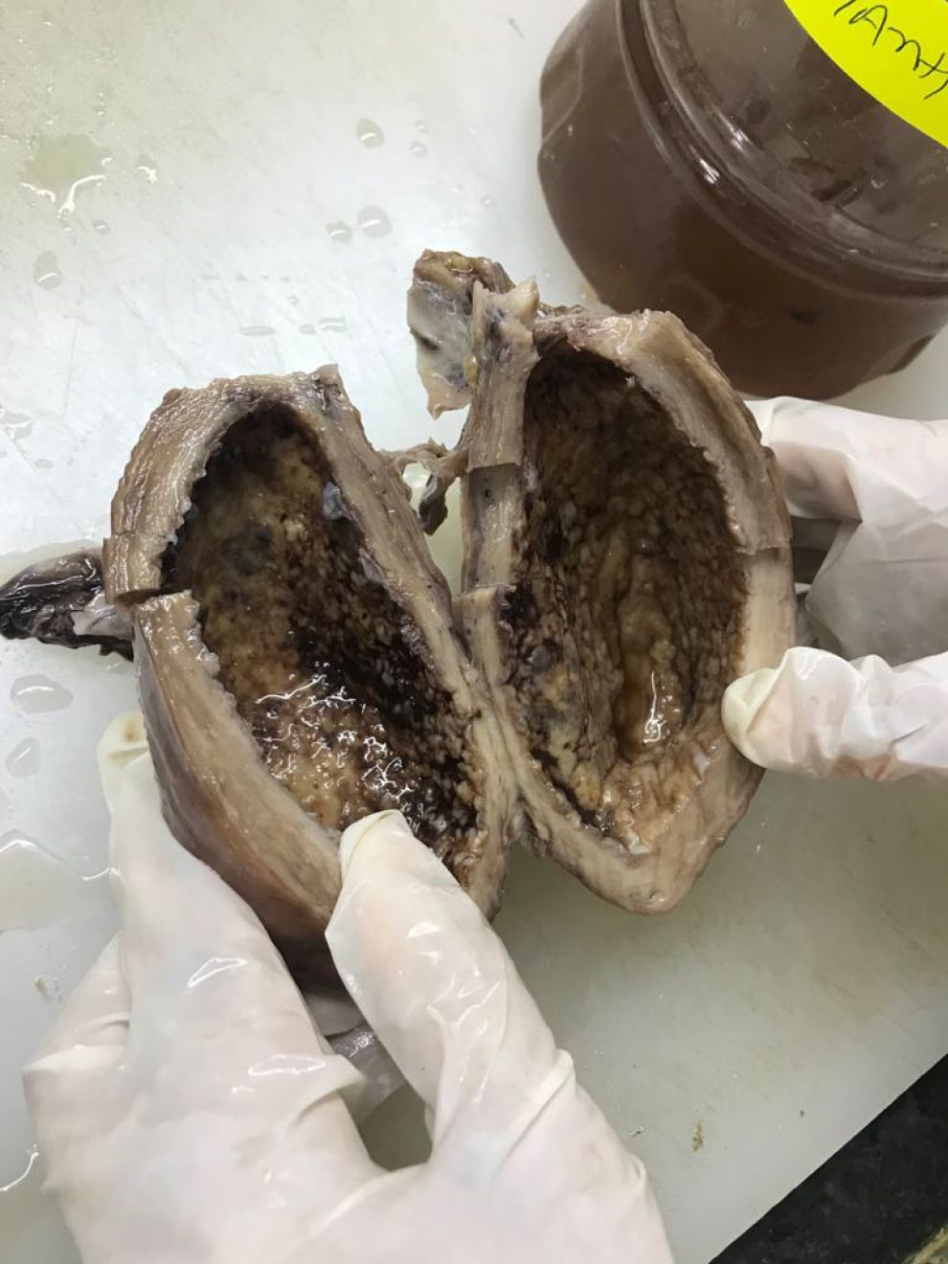
Cut section of uterus showing irregular cavity

**Figure:**
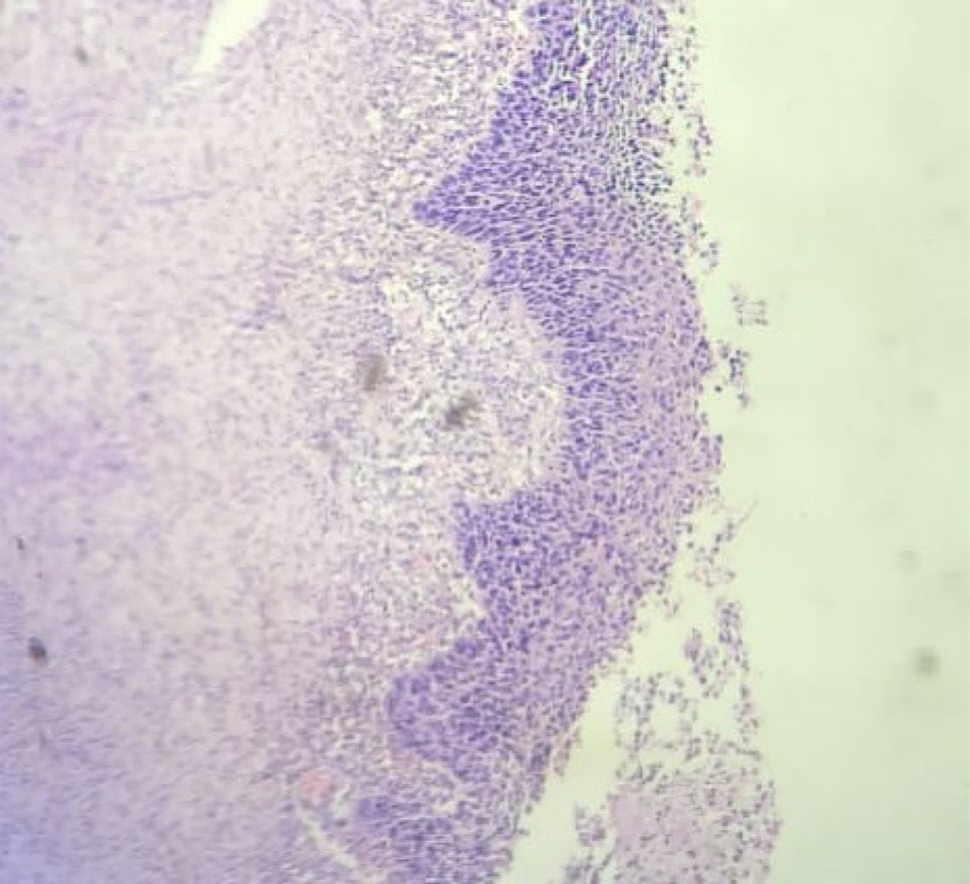
Microscopic view of keratinised epithelium with endometrium beneath

**Figure:**
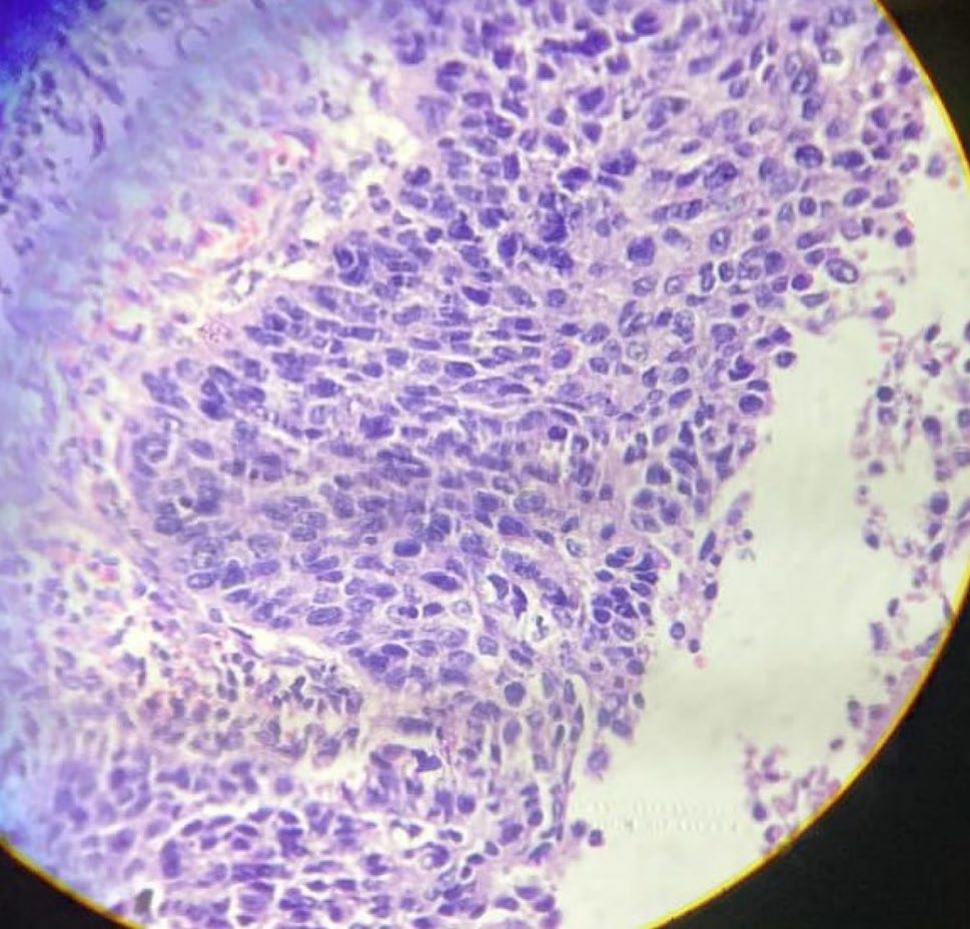
Closer view depicting Dyskaryosis within squamous epithelium overlying endometrial glands

## Supplementary Information

Below is the link to the electronic supplementary material.

Supplementary file1 (DOCX 25 KB)
