# Supplementary material for: Carcinoma Cervix Leading to Ichthyosis Uteri: A Rare Case Report
Source: J Obstet Gynaecol India. 2021 Apr 28;71(5):545–9. doi: 10.1007/s13224-021-01472-3 (PMC8440718; doi:10.1007/s13224-021-01472-3)
Supplement: Supplementary file 1 — Supplementary file1 (DOCX 25 KB) [file 13224_2021_1472_MOESM1_ESM.docx]

**Consent –**

Written informed consent was obtained from the patient for publication of this case report and accompanying images.

**References -**

1.      Fadare O. Dysplastic Ichthyosis uteri-like changes of the entire endometrium associated with a squamous cell carcinoma of the uterine cervix. Diagn Pathol 2006;1:8-11.

2.      Bagga PK, Jaswal TS, Datta U, Mahajan NC. Primary endometrial squamous cell carcinoma with extensive squamous metaplasia and dysplasia. Indian J Pathol Microbiol 2008;51:267-8.

3.      Bewtra C, Xie QM, Hunter WJ, Jurgensen W.Ichthyosis uteri: a case report and review of literature. Arch Pathol Lab Med 2005;129:e124-e125.

4. Marcus SL: Adenoacanthoma of the endometrium: a report of 24 cases and a review of squamous metaplasia. Am J Obstet Gynecol. 1961, 81: 259-267.
